# Limonin, a Component of Immature Citrus Fruits, Activates Anagen Signaling in Dermal Papilla Cells

**DOI:** 10.3390/nu14245358

**Published:** 2022-12-16

**Authors:** Jung-Il Kang, Youn Kyoung Choi, Sang-Chul Han, Hyeon Gyu Kim, Seok Won Hong, Jungeun Kim, Jae Hoon Kim, Jin Won Hyun, Eun-Sook Yoo, Hee-Kyoung Kang

**Affiliations:** 1 Jeju Research Center for Natural Medicine, Jeju National University, Jeju 63243, Republic of Korea; 2 Department of Medicine, School of Medicine, Jeju National University, Jeju 63243, Republic of Korea; 3 Department of Chemistry & Cosmetics, Jeju National University, Jeju 63243, Republic of Korea; 4 Department of Biotechnology, College of Applied Life Science, SARI, Jeju National University, Jeju 63243, Republic of Korea; 5 Subtropical/Tropical Organism Gene Bank, Jeju National University, Jeju 63243, Republic of Korea

**Keywords:** limonin, dermal papilla cells, anagen signaling, autophagy, Wnt/β-Catenin, PI3K/AKT

## Abstract

Hair loss remains a significant problem that is difficult to treat; therefore, there is a need to identify safe natural materials that can help patients with hair loss. We evaluated the hair anagen activation effects of limonin, which is abundant in immature citrus fruits. Limonin increased the proliferation of rat dermal papilla cells (rDPC) by changing the levels of cyclin D1 and p27, and increasing the number of BrdU-positive cells. Limonin increased autophagy by decreasing phosphorylated mammalian target of rapamycin levels and increasing the phospho-Raptor, ATG7 and LC3B. Limonin also activated the Wnt/β-catenin pathway by increasing phospho-β-catenin levels. XAV939, a Wnt/β-catenin inhibitor, inhibited these limonin-induced changes, including induced autophagy, BrdU-positive cells, and cell proliferation. Limonin increased the phosphorylated AKT levels in both two-dimensional cultured rDPC and three-dimensional spheroids. Treatment with the PI3K inhibitor wortmannin inhibited limonin-induced proliferation, and disrupted other limonin-mediated changes, including decreased p27, increased BrdU-positive cells, induced autophagy, and increased ATG7 and LC3B levels. Wortmannin also inhibited limonin-induced cyclin D1 and LC3 expression in spheroids. Collectively, these results indicate that limonin can enhance anagen signaling by activating autophagy via targeting the Wnt/β-catenin and/or PI3K/AKT pathways in rDPC, highlighting a candidate nutrient for hair loss treatment.

## 1. Introduction

Hair has biological functions such as blocking ultraviolet rays and maintaining body temperature [1,2]. Hair is structurally composed of several types of epithelial and mesenchymal cells [3]. Hair growth or loss occurs due to changes in cell differentiation, apoptosis, and cell proliferation in hair follicles that continuously repeat the hair cycle divided into the growth (anagen), regression (catagen), and resting (telogen) phases [3,4]. Dermal papilla cells (DPC) are mesenchymal-derived cells of the hair follicle that exist throughout the hair cycle, which secrete growth factors, including fibroblast growth factor-7, vascular endothelial growth factor, and transforming growth factor-beta, and interact with adjacent matrix cells, and epithelial cells [5,6,7]. In DPC, distinct signaling [Wnt/β-catenin and phosphatidylinositol-3 kinase (PI3K)/AKT] plays a role in cell proliferation and hair-cycle changes. Autophagy is a lysosomal degradation system that maintains cellular homeostasis [8]; its inhibition induces the catagen phase, whereas its activation induces the anagen phase of the hair cycle [9]. Furthermore, Wnt/β-catenin inhibitors inhibit autophagy activation and increase the proliferation of DPC, suggesting an association between Wnt/β-catenin and autophagy [10].

Although hair loss has no effect on human health, a lack of self-confidence in patients with hair loss can cause difficulties in interpersonal relationships and social adjustment. The number of patients with hair loss worldwide is increasing rapidly, regardless of sex or age, with over one million individuals per year seeking treatment for hair loss [11], including hair transplantation or Food and Drug Administration-approved medications (finasteride and minoxidil) [12,13]. Finasteride is an inhibitor of type 2 5α-reductase, which in turn inhibits the production of dihydrotestosterone (DHT), a potent androgen, by inhibiting the conversion of testosterone to DHT [14]. High levels of DHT and androgen receptors have been observed in patients with androgenetic alopecia, suggesting that finasteride may be effective through these actions [14,15]. However, the mechanism of action of minoxidil in improving hair loss remains unclear, with various mechanisms proposed to date, including vasodilation, opening ATP-sensitive potassium channels, activating the Wnt/β-catenin pathway, activating prostaglandin-endoperoxide synthase-1, and inhibiting apoptosis in hair follicle cells [16,17,18,19,20]. However, these two drugs have significant side effects, including temporary effects, irritation of the scalp, and infertility, which poses a challenge to their effectiveness [21,22]. Therefore, there is a high need to identify safe natural products that are effective for hair loss treatment.

Citrus fruits such as juices and teas are commonly consumed as food and contain a variety of bioactive compounds [23]. In particular, compared with mature fruits, immature citrus fruits have a higher content of flavonoids with demonstrated health benefits, and several studies have reported that immature citrus fruits are effective against skin diseases, including atopic dermatitis and aging [24,25,26]. Limonin is a triterpenoid abundant in citrus fruits, and its therapeutic effects have been reported in various human diseases. Limonin showed protective effects against glutamate-induced toxicity in rat cortical cells [27]. Limonin not only induced apoptosis but also enhanced the efficacy of chemotherapeutic agents in human colon adenocarcinoma cells by enhancing the cytotoxicity of doxorubicin at non-toxic concentrations [28,29]. However, there have been no reports of the potential for limonin in promoting hair growth or inhibiting hair loss.

The majority of studies focused on identifying new materials for improving hair loss have used mouse models or hair follicle cultures; however, these models require substantial time and effort compared to studies using cultured cells [30,31]. DPC are onion-shaped, mesenchymal-derived fibroblasts located at the base of the hair follicles and are essential for hair regeneration and hair growth [4,32,33]. Therefore, DPC can be used to screen for a material that can improve hair loss relatively easily before further testing in an animal model. In vivo, DPC exist as three-dimensional (3D) spheroids surrounded by the extracellular matrix [34]. Therefore, the 2D culture of DPC is limited in maintaining a complex environment in vivo. The shape of 3D spheroids of DPC is similar to that found in vivo, but also shows the characteristics of freshly isolated DP signature genes [4,35].

Therefore, to find effective materials for improving hair loss, in this study, we used 2D cultures and 3D spheroids of DPC to investigate whether limonin has inductive effects by activating anagen signaling. Specifically, we focused on the effects of limonin on cell proliferation and autophagy. To further explore the underlying mechanisms, we also examined the effects on cell cycle and proliferation regulators, including p27 (also known as KIP1), which is a cyclin-dependent kinase inhibitor that regulates cell proliferation through cell cycle inhibition [36]. Minoxidil and various natural extracts are known to regulate hair growth by targeting p27 [37,38]. In addition, p27 transcription is downregulated by AKT [39]. We further explored the effect on PI3K/AKT as a representative signaling pathway involved in the survival and proliferation of various cells, including DPC [40,41]. In addition, PI3K/AKT signaling has been reported to play an essential role in hair follicles regeneration [42]. Together, these findings can provide a basis for further research and development of expanding the applications of limonin, as a nutrient found in immature citrus fruits, in the treatment of hair loss.

## 2. Materials and Methods

### 2.1. Preparation of Immature Citrus Fruit Extract

The peel of immature citrus fruits native to Jeju Island was washed with distilled water and dried. The sample was homogenized using a grinder and extracted with 70% ethanol at 2000 rpm and 25 °C for 2 h (WIS 20R, WITEG, Wertheim, Germany). After extraction and filtration, the sample was concentrated using a vacuum evaporator (Hei-VAP Advantage, Heldolph, Germany) and lyophilized to a powder.

### 2.2. Cell Culture

Immortalized rat vibrissa DPC (rDPC) were donated by the Skin Research Institute of the Amore Pacific R&D Center (Yongin, Korea). The cells were cultured in Dulbecco’s modified Eagle medium (DMEM; HyClone, Logan, UT, USA) containing 1% antibiotics (Gibco Life Technologies, Grand Island, NY, USA) and 10% heat-inactivated fetal bovine serum (FBS; HyClone). The cells were maintained at 37 °C in a humidified atmosphere with 5% CO_2_ and sub-cultured every three days.

### 2.3. Proliferation Assay

The proliferation of rDPC was measured using the water-soluble tetrazolium (WST) assay kit (EZ-Cytox; Daeil Lab Service, Seoul, Korea) according to the manufacturer’s protocol. For 2D culture, rDPC (2000 cells/well) were seeded in 96-well plates in DMEM containing 1% FBS (low-serum condition) for 24 h. The cells were stimulated with immature citrus fruit extract (0.1, 1, 10, and 100 μg/mL), rhoifolin (0.1, 1, and 10 μM; Sigma-Aldrich, St. Louis, MO, USA), hesperidin (0.1, 1, and 10 μM; Sigma-Aldrich), and limonin (0.1, 1, and 10 μM; Sigma-Aldrich) for 72 h. To investigate whether the PI3K/AKT or Wnt/β-catenin pathway affects the limonin-mediated proliferation of 2D rDPC, the cells were pretreated with 10 nM of the PI3K/AKT inhibitor wortmannin (Invitrogen, Carlsbad, CA, USA) or 10 μM of the Wnt/β-catenin inhibitor XAV939 (Tocris Bioscience, Bristol, UK) for 2 h, and then treated with limonin for 72 h. To investigate the effects of limonin on 3D cultured rDPC (3D spheroids), 2D rDPC were cultured in low-attachment 96-well plates for 48 h and treated with limonin for 72 h after 3D spheroid generation. To investigate whether the PI3K/AKT pathway affects limonin-mediated proliferation of 3D spheroids, 3D spheroids were stimulated with 10 nM of wortmannin for 2 h and then treated with or without limonin for 72 h. WST dye (10 μL/well) was added to a 96-well plate and incubated for 3 h. Absorbance (450 nm) was measured using a Versamax microplate reader (Molecular Devices, Sunnyvale, CA, USA). The experiment was performed thrice, and the changes in the drug-treated groups were compared with the average absorbance value of the dimethyl sulfoxide (DMSO; Sigma-Aldrich)-treated control group. All chemicals were dissolved in DMSO. The final concentration of DMSO used in the experiments is at 0.2% or less.

### 2.4. Western Blot Analysis

The 2D cultured rDPC and 3D spheroids were treated with or without 10 μM of limonin or 10 μM of minoxidil (Sigma-Aldrich) as a positive control for 24 h or 0–6 h. In some cases, cells were treated with wortmannin (10 nM) or XAV939 (10 μM) for 2 h, followed by treatment with limonin (10 μM) for 24 h, as described above. The cells were washed with Dulbecco’s phosphate-buffered saline (PBS; Welgene, Daegu, Korea) and lysed using the PRO-PREP^TM^ protein extraction solution (iNtRON Biotechnology, Seoul, Korea) for 1 h at 4 °C. Proteins (20 μg) were separated using sodium dodecyl sulfate-polyacrylamide gel electrophoresis and transferred onto polyvinylidene fluoride membranes (Bio-Rad, Hercules, CA, USA). The membranes were blocked with 5% non-fat dry milk for 90 min and incubated with primary antibodies (Table 1) for 18 h at 4 °C. Membranes were washed and incubated with horseradish peroxidase-labeled secondary antibodies for 1 h at room temperature. The protein bands were visualized on an X-ray film (Agfa-Gevaert, Mortsel, Belgium) using a Westar Nova 2.0 ECL solution (Cyanagen, Bologna, Italy).

### 2.5. Immunofluorescence Staining

The 2D rDPC were cultured in Nunc™ Lab-Tek™ II Chamber Slides™ (Thermo Fisher Scientific, Waltham, MA, USA) in a low-serum condition for 24 h. The cells were stimulated with or without rhoifolin, hesperidin, limonin, or minoxidil for 1 h or 24 h. In some experiments, the cells were treated with 10 nM of wortmannin or 10 μM of XAV939 for 2 h and then treated with or without limonin (10 μM) for 24 h. For 5′-bromo-2′-deoxyuridine (BrdU) incorporation, the cells were labeled with 10 μM of BrdU for 3 h, fixed with 4% paraformaldehyde (PFA) for 15 min, and permeabilized with PBS containing 0.1% Triton^TM^ X-100 (permeable buffer) for 15 min. The cells were denatured with 2 M HCl containing 0.5% Triton^TM^ X-100 for 30 min and neutralized with 0.1 M sodium borate for 30 min. For immunofluorescence staining, the cells were fixed with 4% PFA for 15 min and subjected to a permeable buffer for 15 min. The cells were incubated with blocking buffer (PBST containing 1% bovine serum albumin and 22.52 mg/mL glycine; Sigma-Aldrich) for 1 h, followed by incubation with primary antibodies (phospho-ser675- β-catenin, LC3B, BrdU, or α-tubulin) for 18 h at 4 °C. The cells were washed and incubated with Alexa Fluor^®^ 488- or Alexa Fluor^®^ 594-conjugated secondary antibodies (Invitrogen) for 1 h before being covered with Vectastain mounting medium (Vector Laboratories, Burlingame, CA, USA) to visualize nuclei. The cellular localization of proteins was obtained using a FluoView^®^ FV1200 confocal microscope (Olympus, Tokyo, Japan).

### 2.6. Statistical Analysis

Data are expressed as the mean ± standard deviation of at least three experiments. The differences between experimental groups were statistically evaluated using Student’s *t*-test. GraphPad Prism 7 (GraphPad Software, San Diego, CA, USA) was used for statistical analyses. A *p*-value < 0.05 was considered statistically significant.

## 3. Results

### 3.1. Immature Citrus Fruit Peel-Derived Limonin Increases the Proliferation of 2D Dermal Papilla Cells

**Figure 1:**
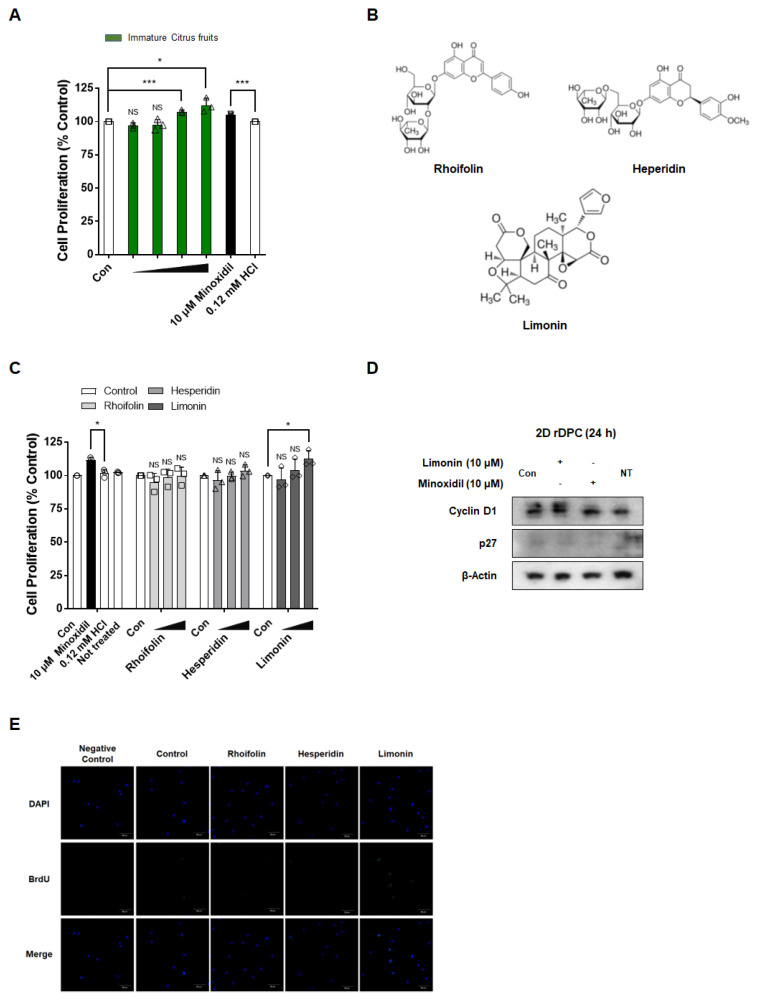
Limonin, a component of immature citrus fruits, induces the proliferation of two-dimensional (2D) rDPC. (**A**) Effects of immature citrus fruit peel extract on the proliferation of 2D rDPC. (**B**) Structure of the components (rhoifolin, hesperidin, and limonin) from immature citrus fruits. (**C**) Effects of rhoifolin, hesperidin, and limonin on the proliferation of 2D rDPC. (**D**) Representative images of Western blots for cell cycle-related proteins cyclin D1 and p27 after treatment with limonin and minoxidil for 24 h. (**E**) Confocal microscopy images for changes in the number of BrdU-positive cells after treatment with rhoifolin, hesperidin, and limonin for 24 h. Data are presented as the mean ± standard deviation of three independent experiments. * *p <* 0.05, *** *p <* 0.001 vs. vehicle-treated control. Scale bars, 50 μm. Con, vehicle-treated control; 0.12 mM HCl, vehicle for minoxidil; NS, not significant.

When 2D rDPC were exposed to immature citrus fruit extract at 0.1, 1, 10, and 100 μg/mL for 72 h, proliferation was enhanced in a concentration-dependent manner by 97.0 ± 2.1%, 97.8 ± 4.0%, 107.5 ± 1.3% (*p* < 0.001), and 112.3 ± 5.3% (*p* < 0.05), respectively (Figure 1A). Minoxidil, as a positive control, also effectively increased the proliferation of 2D rDPC (105.0 ± 0.8%; *p* < 0.001). To investigate which components of immature citrus fruits induced the proliferation of rDPC, we evaluated the proliferative effects of individual components (rhoifolin, hesperidin, and limonin) from immature citrus fruits on 2D rDPC. Limonin increased the proliferation of 2D rDPC in a dose-dependent manner by 97.1 ± 8.6%, 104.1 ± 8.1%, and 112.4 ± 6.3% (*p* < 0.05) at 0.1, 1, and 10 μg/mL, respectively (Figure 1C). However, rhoifolin and hesperidin did not affect the proliferation of the cells.

To explore the mechanism of limonin-induced cell proliferation, the levels of the cell cycle-related proteins cyclin D1 and p27 were investigated after limonin (10 μM; the most effective concentration) treatment. As shown in Figure 1D, compared with those of the DMSO-treated control group, limonin increased the levels of cyclin D1 and decreased the levels of p27.

Next, we validated whether limonin promoted the proliferation of rDPC by a BrdU incorporation assay. The number of BrdU-positive cells was not changed by rhoifolin and hesperidin but was increased in the limonin-treated group compared to the vehicle-treated group (Figure 1E). Our findings suggest that limonin, a component of immature citrus fruit peels, stimulates the proliferation of 2D rDPC by regulating cell cycle-related proteins.

### 3.2. Limonin Activates Autophagy in 2D Dermal Papilla Cells

**Figure 2:**
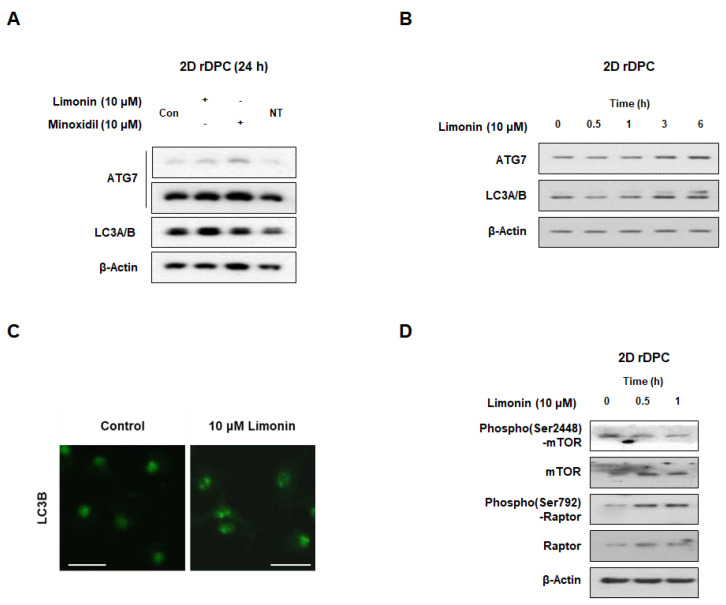
Limonin activates autophagy pathways in two-dimensional cultured rDPC. (**A**) Representative images of Western blot for autophagy-related proteins ATG7 and LC3A/B after treatment with limonin and minoxidil (positive control) for 24 h. (**B**) Representative images of Western blots for ATG7 and LC3A/B after treatment with limonin for 0–6 h. (**C**) Confocal microscopy images for changes in the number of LC3B puncta after treatment with limonin for 24 h. (**D**) Representative images of Western blots for upstream regulators of autophagy, phospho(Ser2448)-mTOR and phospho(Ser792)-Raptor, after treatment with limonin for 0, 0.5, and 1 h. Scale bars, 50 μm. Con, vehicle-treated control; NT, not-treated.

We next investigated the effect of limonin on autophagy in 2D rDPC. As shown in Figure 2A, limonin increased the levels of ATG7 and LCB compared with those in the control group. In addition, ATG7 and LCB levels increased after 3 h of limonin treatment (Figure 2B). Limonin also increased the number of LC3 puncta (Figure 2C). When 2D rDPC were treated with 10 μM limonin for 0.5 and 1 h, the phosphorylation levels of mammalian target of rapamycin (mTOR) at Ser^2448^ decreased and the phosphorylation levels of raptor (Ser^792^) increased (Figure 2D). These data indicated that limonin activates autophagy by inhibiting mTORC1 in 2D cultured rDPC.

### 3.3. Limonin Stimulates Autophagy by Activation of Wnt/β-Catenin Signaling in 2D Dermal Papilla Cells

**Figure 3:**
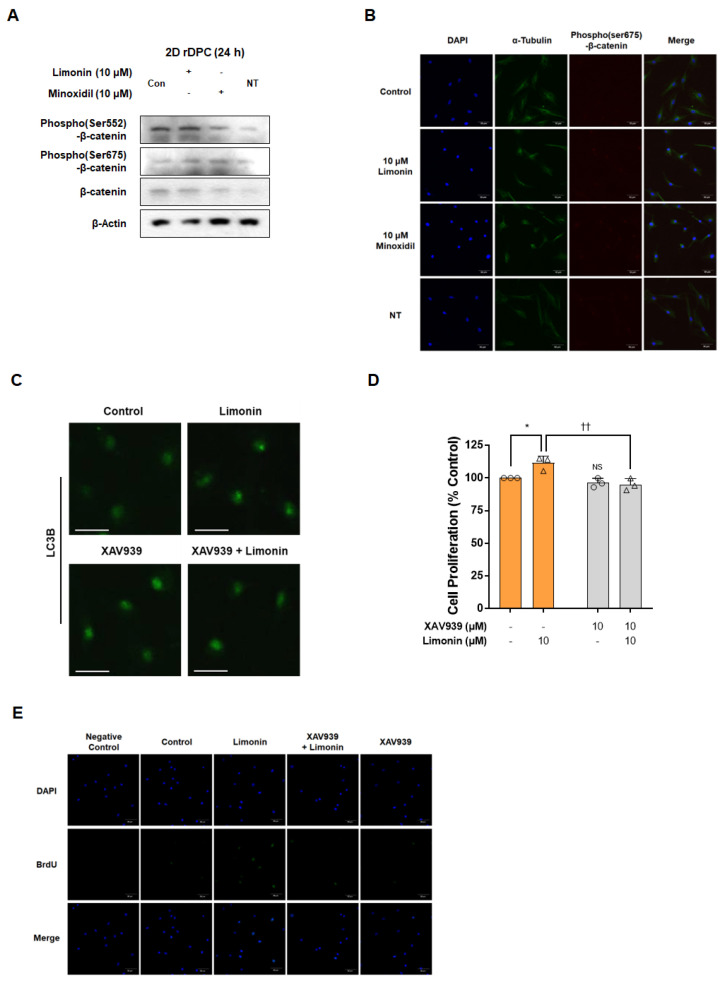
Limonin induces cell proliferation via activation of the Wnt/β-catenin pathway in two-dimensional (2D) cultured rDPC. (**A**) Representative images of Western blot for Wnt/β-catenin-related proteins phospho(Ser552)-β-catenin and phospho(Ser675)-β-catenin after treatment with limonin and minoxidil for 24 h. (**B**) Confocal microscopy images for changes in the cellular localization of phospho(Ser675)-β-catenin after treatment with limonin and minoxidil for 1 h. (**C**) Confocal microscopy images of changes in the number of LC3B puncta after treatment with limonin for 24 h in the absence or presence of XAV939. (**D**) Changes in the proliferation of 2D rDPC after treatment with limonin for 3 days with or without XAV939. (**E**) Confocal microscopy images for changes in the number of BrdU-positive cells after treatment with limonin for 24 h in the absence or presence of XAV939. * *p* < 0.05 vs. vehicle-treated control. ^††^ *p* < 0.01 vs. limonin alone. Scale bars, 50 μm. Con, vehicle-treated control; NT, not treated; NS, not significant.

When 2D cultured rDPC were treated with 10 μM of limonin for 24 h, the levels of phospho(Ser552 and Ser675)-β-catenin were higher than those in the control group (Figure 3A). The positive control, minoxidil, also increased the expression levels of phospho(Ser552 and Ser675)-β-catenin compared with those of the untreated control group. Immunofluorescence staining showed that the level of phospho(Ser675)-β-catenin in the nucleus increased after treatment with both limonin (10 μM) and minoxidil (10 μM) for 24 h (Figure 3B). These data indicated that limonin stimulated the nuclear translocation of phospho(Ser675)-β-catenin.

To further confirm whether stimulation of the Wnt/β-catenin pathway modulates limonin-induced 2D rDPC proliferation and autophagy induction, experiments were performed using the Wnt/β-catenin signaling inhibitor XAV939. As shown in Figure 3C, inhibition of Wnt/β-catenin signaling by XAV939 treatment in 2D rDPC reduced the formation of limonin-induced LC3B puncta. The WST assay showed that limonin treatment alone increased the proliferation of 2D rDPC (111.5 ± 5.3%; *p* < 0.05), whereas combined treatment with limonin (10 μM) and XAV939 (10 μM) attenuated the limonin-induced proliferation of 2D rDPC (94.9 ± 4.6%; *p* < 0.05) (Figure 3D). Similarly, the increased number of BrdU-positive cells induced by limonin was suppressed by XAV939 pre-treatment (Figure 3E), suggesting that activation of the Wnt/β-catenin pathway by limonin is necessary to increase autophagy and ultimately induce the proliferation of 2D rDPC.

### 3.4. Inhibition of the PI3K/AKT Pathway Attenuated Limonin-Mediated Proliferation by Activation of the Autophagy Pathway in 2D Dermal Papilla Cells

**Figure 4:**
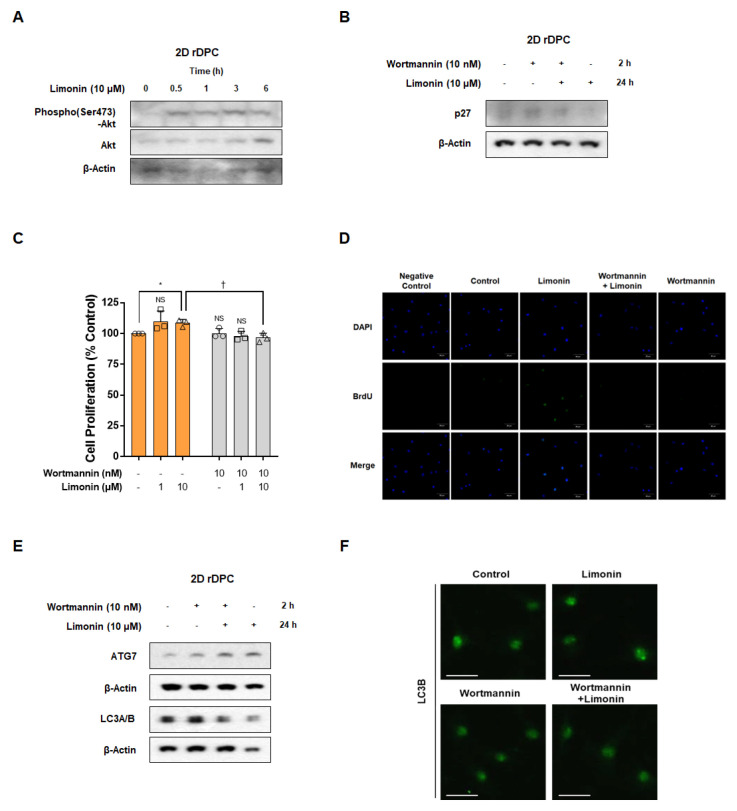
Limonin induces cell proliferation through activation of the autophagy pathway via PI3K/AKT signaling in two-dimensional (2D) cultured rDPC. (**A**) Representative images of Western blots for phospho(Ser473)-AKT after treatment with limonin for 0–6 h. (**B**) Representative images of Western blots for p27 after treatment with limonin for 24 h in the absence or presence of wortmannin. (**C**) Changes in the proliferation of 2D dermal papilla cells after treatment with limonin for 3 days with or without wortmannin. (**D**) Confocal microscopy images for changes in the number of BrdU-positive cells after treatment with limonin for 24 h with or without wortmannin. (**E**) Representative images of Western blots for ATG7 and LC3A/B after treatment with limonin for 24 h with or without wortmannin. (**F**) Confocal microscopy images of changes in the number of LC3B puncta after treatment with limonin for 24 h with or without wortmannin. * *p* < 0.05 vs. vehicle-treated control. ^†^ *p* < 0.05 vs. limonin alone. Scale bars, 50 μm. NS, not significant.

Limonin stimulated AKT phosphorylation after 0.5–6 h (Figure 4A). We investigated whether the reduction in p27 by limonin correlated with limonin-mediated AKT activity. Wortmannin, a PI3K/AKT inhibitor, restored the limonin-induced reduction in p27 levels (Figure 4B). Limonin treatment alone (10 μM) stimulated the proliferation of 2D rDPC (108.5 ± 2.9%; *p* < 0.05), whereas pre-treatment with wortmannin (10 nM) significantly attenuated the limonin-induced proliferation of 2D rDPC (96.6 ± 3.8%; *p* < 0.05) (Figure 4C). In addition, wortmannin inhibited the increase in the number of BrdU-positive cells induced by limonin treatment, suggesting that limonin activates the PI3/AKT pathway to promote the proliferation of 2D rDPC (Figure 4D). Next, we examined whether AKT signaling affects autophagy. The limonin-induced increase in ATG7 and LC3B expression was inhibited by pre-treatment with wortmannin (Figure 4E). In addition, wortmannin decreased limonin-induced LC3B puncta (Figure 4F). These results suggested that limonin-induced activation of PI3K/AKT is important for cell cycle regulation and autophagy induction, and ultimately affects the proliferation of 2D rDPC.

### 3.5. Limonin Promotes the Proliferation of 3D Spheroids through Activation of PI3K/AKT Pathway-Mediated Autophagy

**Figure 5:**
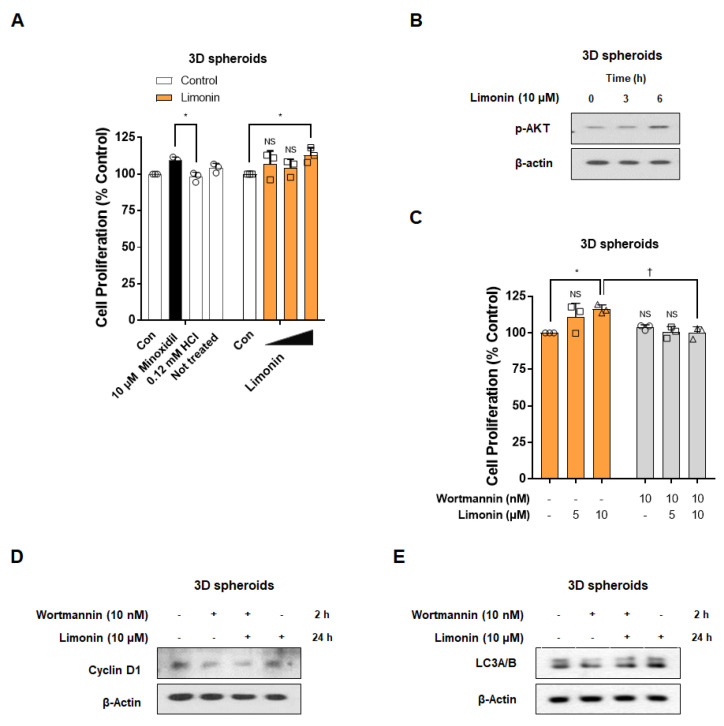
Limonin induces cell proliferation through alterations in the autophagy pathway by activation of the PI3K/AKT pathway in three-dimensional (3D) spheroids. (**A**) Effects of limonin on the proliferation of 3D spheroids. (**B**) Representative images of Western blots for phospho(Ser473)-AKT in 3D spheroids treated with limonin for 0–6 h. (**C**) Changes in the proliferation of 3D spheroids after treatment with limonin for 3 days with or withoutwortmannin. (**D**) Representative images of Western blots for p27 in 3D spheroids treated with limonin for 24 h with or without wortmannin. (**E**) Representative images of Western blots for LC3A/B in 3D spheroids treated with limonin for 24 h with or without wortmannin. * *p <* 0.05 vs. vehicle-treated control. ^†^ *p <* 0.05 vs. limonin alone. Con, vehicle-treated control; 0.12 mM HCl, vehicle for minoxidil; NS, not significant.

Similar to the effects observed in 2D culture conditions, limonin stimulated the proliferation of 3D spheroids by 106.7 ± 9.2%, 104.3 ± 5.8%, and 112.9 ± 5.1% (*p* < 0.05) at 0.1, 1, and 10 μM, respectively. Minoxidil (10 μM, positive control) also significantly increased the proliferation of 3D spheroids (109.4 ± 2.3%; *p* < 0.05) compared to that in the vehicle-treated group (98.0 ± 3.3%) (Figure 5A). We next examined the efficacy of limonin on AKT phosphorylation, cell cycle protein expression, and autophagy induction in 3D spheroids. Stimulation with limonin (10 μM) in 3D spheroids increased the phosphorylation levels of AKT after 3–6 h (Figure 5B). Therefore, we investigated whether activation of the PI3K/Akt pathway by limonin is essential for cell proliferation in 3D spheroids, similar to 2D rDPC. Wortmannin (10 nM) pre-treatment significantly reduced cell proliferation compared to treatment with limonin (10 μM) alone (Figure 5C). Limonin increased the expression of cyclin D1, a cell cycle-related protein, and LC3B, an autophagy-related protein, in 3D spheroids, which was suppressed by PI3K/AKT inhibition using wortmannin (Figure 5D,E). These results suggest that activation of the PI3K/AKT pathway by limonin is essential for proliferation in 3D spheroids, similar to 2D rDPC.

## 4. Discussion

Recently, there has been increased research interest in finding natural materials to develop new treatments for hair loss. The present study showed that limonin, a component of immature citrus, could promote the proliferation of 2D rDPC and 3D spheroids by activating anagen signaling.

Several studies have compared mature and immature citrus fruits, with some reports showing that immature citrus fruits with a relatively high content of flavonoids show superior antioxidant and antiaging effects [24,43]. In this study, immature citrus fruit extract significantly increased the proliferation of 2D rDPC. Limonin (10 μM) showed a proliferative effect similar to that of minoxidil, a positive control, in 2D rDPC, and the same trend was observed in 3D spheroids, whereas other citrus components, rhoifolin and hesperidin, had no effect on rDPC proliferation. This result was confirmed based on the incorporation of BrdU, an analog of the nucleoside thymidine [44].

Cell cycle-related proteins and the cell cycle change with cell proliferation and apoptosis in mammalian cells [45,46]. Cyclin D1 increases the level of phospho-pRB in the G1 phase and promotes cell cycle progression, whereas cyclin-dependent kinase (CDK) inhibitors such as p27 inhibit cyclin/CDK activation and cell cycle progression [45,47]. Limonin-induced increases in cyclin D1 levels or decreases in p27 levels were observed in 2D rDPC and 3D spheroids. A previous study in mice showed that p27 expression was lower in the intermediate hair follicles than in the small hair follicles, and the number BrdU-positive cells was increased in the intermediate hair follicles compared with that in the small hair follicles [48]. This is similar to the effects of limonin in 2D rDPC, suggesting that limonin may affect not only the proliferation of hair follicle cells but also the size of hair follicles by regulating cell cycle-related proteins.

Cell proliferation and death in hair follicles are driven by the modification of various pathways, which change during the transition of the hair cycle and contribute to hair growth and loss. In this study, we investigated the mechanisms of action, including autophagy, PI3K/AKT signaling, and Wnt/β-catenin signaling, associated with hair growth or the hair cycle in 2D rDPC and 3D spheroids to elucidate whether limonin activates anagen signaling. Autophagy is essential for the maintenance of cellular homeostasis [8]. Activation of autophagy has been reported to be related to maintenance of the anagen phase of the hair cycle and inhibition of premature hair follicle regression [9,49]. LC3 plays an essential role during the initial stages of autophagy. Binding of LC3 to phosphatidylethanolamine for autophagosome formation requires the action of conjugation machinery, including ATG7 and ATG3 [50]. In a scalp hair follicle organ culture model, the expression of LC3B increased in the anagen phase rather than in the catagen phase of the hair cycle [9]. Recently, changes in autophagy-related molecules during the hair cycle and the role of autophagy in hair growth have been revealed [9,10,51]. The level of LC3B, an autophagy-related protein, is expressed in the matrix cells of cultured human hair follicles and decreases in the catagen phase compared to the anagen phase, while inhibition of ATG5 leads to catagen progression [9]. Small molecules that promote the transition from telogen to anagen in mice activate autophagy, and their effects are blocked by autophagy inhibitors, suggesting a correlation between hair growth and autophagy [51]. The conversion of LC3A to LC3B by ATG7 induces the formation of autophagosomes, which is determined by the number of LC3B puncta [52]. We found that limonin increased the LC3B and ATG7 levels and the number of LC3 puncta in the 2D rDPC and LC3B levels in 3D spheroids, suggesting that limonin activates autophagy. The mTOR complex 1 signaling pathway (mTORC1, consisting of mTOR, Raptor, GβL, and Deptor) plays an important role in autophagy regulation [53]. Raptor binds to mTOR and contributes to its action as a negative regulator of autophagy [8,54]. Limonin induced changes in mTORC1 signaling, such as decreased levels of phospho(Ser2448)-mTOR and increased levels of phospho(Ser792)-Raptor, eventually inducing autophagy in 2D rDPC.

The Wnt/β-catenin pathway, which interacts with various signaling pathways, is a major factor in hair growth and regeneration [18,55]. Activation of the autophagy pathway is associated with increased cell proliferation through modification of the Wnt/β-catenin pathway in rDPC [10]. The Wnt/β-catenin pathway is involved in hair growth initiation, morphogenesis, and regeneration [55,56]. DPC requires the Wnt/β-catenin pathway to maintain hair-inducing activity [57]. After ligand–receptor binding, β-catenin is stabilized/accumulated by an increase in phospho(Ser9)-GSK3β and is then translocated to the nucleus to regulate target gene expression [58]. Kinases such as PKA or PKB also induce an increase in the levels of phospho(Ser 9)-GSK3β and phospho(Ser552 or Ser675)-β-catenin, which contributes to the stabilization of β-catenin and its subsequent nuclear translocation [59,60]. Minoxidil, a drug that is commonly used to improve hair loss, induces upregulation of nuclear β-catenin in human DPC and increases the level of Wnt target genes (*Axin2* and *Lef-1*), thereby prolonging the anagen phase in mice [18]. Dickkopf 1 (DKK-1), a Wnt antagonist, exhibits higher levels in the balding scalp than in the non-balding scalp, and promotes apoptosis in outer root sheath cells and catagen progression in hair follicles [61,62]. This suggests that activation of the Wnt/β-catenin pathway is essential for extension of the anagen phase by inhibiting apoptosis in hair follicle cells. Indeed, we found that the phosphorylation and nuclear localization of β-catenin were higher in cells treated with limonin than in controls, and the increase in limonin-mediated proliferation and BrdU-positive cells was prevented by inhibition of the Wnt/β-catenin pathway. Moreover, the increase in the number of LC3B puncta caused by limonin in 2D rDPC was inhibited in the presence of XAV939. These results suggest that limonin activates the autophagy pathway via Wnt/β-catenin signaling to induce anagen signaling in rDPC.

The PI3K/AKT signaling pathway is a crucial role in several cellular activities, including cell survival and cell death [63]. Specific inhibitors of the PI3K/AKT pathway were reported to disrupt hair follicle regeneration from a mixture of epidermal stem cells and skin-derived precursors [42]. The promotion of length growth in scalp hair follicles by minoxidil was found to be accompanied by activation of AKT in human DPC [64]. These previous results suggest that changes in the PI3K/AKT pathway are involved in the promotion of hair regeneration and proliferation of rDPC. We observed that limonin induced an increase in phospho(Ser473)-AKT levels in 2D rDPC, whereas treatment with wortmannin, a PI3K/AKT-specific inhibitor, suppressed the limonin-induced proliferation of 2D rDPC and 3D spheroids, indicating that limonin may induce the proliferation of rDPC by targeting the PI3K/AKT pathway. The role of the PI3K/AKT pathway in limonin-induced proliferation was also supported by changes in cell cycle-related proteins (p27 or cyclin D1) observed in 2D rDPC and 3D spheroids. In contrast, inhibition of PI3K/AKT using wortmannin suppressed the increase in the number of LC3B puncta caused by limonin in 2D rDPC.

Together, these results indicate that limonin induces the activation of autophagy by targeting the PI3K/AKT pathway and promoting the proliferation of rDPC. However, our results also suggest that limonin may inhibit the PI3K/AKT pathway by decreasing the level of phospho(Ser2448)-mTOR. Therefore, the associations between PI3K/AKT activation and the upstream regulator (phospho-mTOR) of autophagy should be elucidated through further studies. Nevertheless, these results suggest that alteration of autophagy via the PI3K/AKT or Wnt/β-catenin pathways by limonin is associated with the activation of anagen signaling in hair follicle cells.

## 5. Conclusions

In this study, we confirmed that the immature citrus component limonin has proliferation-inducing potential of dermal papilla cells, which is related to the modification of autophagy via the PI3K/AKT and/or Wnt/β-catenin pathways. Collectively, our study suggests that limonin could be developed as a useful treatment for hair loss by activating anagen signaling in DPC.

## Figures and Tables

**Table 1:** List of antibodies used for immunoblotting.

Antibodies	Species	Dilution	Supplier
Cyclin D1	Mouse	1:1000	BD Biosciences
p27	Rabbit	1:1000	Santa Cruz
ATG7	Rabbit	1:1000	Cell Signaling
LC3A/B	Rabbit	1:1000	Cell Signaling
LC3B	Rabbit	1:1000	Abcam
BrdU	Mouse	1:200	Thermo Fisher Scientific
Phospho(Ser2448)-mTOR	Rabbit	1:1000	Cell Signaling
mTOR	Rabbit	1:1000	Cell Signaling
Phospho(Ser792)-Raptor	Rabbit	1:1000	Cell Signaling
Raptor	Rabbit	1:1000	Cell Signaling
Phospho(Ser473)-Akt	Rabbit	1:1000	Cell Signaling
Akt	Rabbit	1:1000	Cell Signaling
Phospho(Ser552)-β-catenin	Rabbit	1:1000	Cell Signaling
Phospho(Ser675)-β-catenin	Rabbit	1:1000	Cell Signaling
β-catenin	Rabbit	1:1000	Cell Signaling
α-Tubulin	Mouse	1:1000	Santa Cruz
β-Actin	Mouse	1:1000	Santa Cruz

## Data Availability

All the data needed to evaluate the conclusions are present in the paper, further inquiries can be directed to the corresponding authors.
